# Gaining the Upper Hand: Evidence of Vertical Asymmetry in Sex-Categorisation of Human Hands

**DOI:** 10.5709/acp-0164-8

**Published:** 2014-12-31

**Authors:** Genevieve L. Quek, Matthew Finkbeiner

**Affiliations:** Department of Cognitive Science, Macquarie University, Sydney Perception in Action Research Centre (PARC), Macquarie University, Sydney ARCRC Centre of Excellence in Cognition and its Disorders (CCD)

**Keywords:** vertical asymmetry, upper visual field, lower visual field, attention, sex-categorisation, hands

## Abstract

Visual perception is characterised by asymmetries arising from the brain’s preferential response to particular stimulus types at different retinal locations. Where the lower visual field (LVF) holds an advantage over the upper visual field (UVF) for many tasks (e.g., hue discrimination, contrast sensitivity, motion processing), face-perception appears best supported at above-fixation locations (Quek & Finkbeiner, 2014a). This finding is consistent with Previc’s (1990) suggestion that vision in the UVF has become specialised for object recognition processes often required in ”extrapersonal” space. Outside of faces, however, there have been very few investigations of vertical asymmetry effects for higher-level objects. Our aim in the present study was, thus, to determine whether the UVF advantage reported for face-perception would extend to a nonface object – human hands. Participants classified the sex of hand images presented above or below central fixation by reaching out to touch a left or right response panel. On each trial, a briefly presented spatial cue captured the participant’s spatial attention to either the location where the hand was about to appear (valid cue) or the opposite location (invalid cue). We observed that cue validity only modulated the efficiency of the sex-categorisation response for targets in the LVLVF and not the UVF, just as we have reported previously for face-sex categorisation (Quek & Finkbeiner, 2014a). Taken together, the data from these studies provide some empirical support for Previc’s (1990) speculation that object recognition processes may enjoy an advantage in the upper-hemifield.

## Introduction

The notion of vertical asymmetry in visual perception is by no means new. We have known for some time, for example, that the lower visual field (LVF) exhibits an advantage over the upper visual field (UVF) in terms of contrast sensitivity (Cameron, Tai, & Carrasco, 2002; Carrasco, Penpeci-Talgar, & Cameron, 2001; Carrasco, Williams, & Yeshurun, 2002; T. Liu, Heeger, & Carrasco, 2006; Skrandies, 1987), visual acuity (Skrandies, 1987), spatial resolution (Carrasco et al., 2002; Talgar & Carrasco, 2002), hue discrimination (Levine & McAnany, 2005), and motion processing (Rezec & Dobkins, 2004; Levine & McAnany, 2005). By contrast, the UVF appears to enjoy an advantage on tasks involving visual search (Chaiken, Corbin, & Volkmann, 1962; Fecteau, Enns, & Kingstone, 2000; Previc & Blume, 1993), perception of depth (Levine & McAnany, 2005), and object recognition (Chambers, McBeath, Schiano, & Metz, 1999). Such perceptual asymmetries may in part reflect underlying physiological differences between the upper and lower retinae and subsequent visual pathways. For example, better task performance for LVF stimuli could relate to greater cone and ganglion cell densities in the superior part of the retina on which information presented in the LVF falls (Perry & Cowey, 1985). Similarly, studies with non-human primates have suggested that slightly more neural tissue in lateral geniculate nucleus corresponds to representations of the LVF than the UVF (Connolly & Van Essen, 1984), V1 (Tootell, Switkes, Silverman, & Hamilton, 1988; Van Essen, Newsome, & Maunsell, 1984), and MT (Maunsell & Van Essen, 1987). In humans, the same Gabor stimuli have been shown to evoke a larger volume of activity in early visual cortex when presented in the LVF compared to the UVF (T. Liu et al., 2006).

One intriguing possibility is that the differences in visual capabilities between the UVF and LVF relate to their respective associations with far and near space. Previc (1990) proposed that the capabilities of each vertical hemifield have become specialised to support the visual perception functions most often required in that region of space. According to Previc, visual perception in the LVF has evolved to facilitate visuomotor coordination required in near or *peripersonal* space, the region in which we reach towards and manipulate objects. Conversely, the UVF is linked to the visual search and recognition mechanisms most often required in far or *extrapersonal space*, the region in which we typically search for and discriminate objects and people. While Previc’s functional specialisation account of the vertical anisotropy in visual perception remains contentious (Bracewell, 1990; Karim & Kojima, 2010; Williams, 1990), mounting evidence of an upper-hemifield advantage in face-perception has provided some support for this view (for a review, see Quek & Finkbeiner, 2014a). For example, participants in a study by Felisberti and McDermott (2013) recognised previously seen faces better if they had initially encoded the faces in the upper-hemifield rather than the lower-hemifield. Others have shown that regions such as the medial prefrontal cortex (MPFC), left fusiform face area (FFA), and left occipital face area (OFA) are activated earlier by faces presented in the UVF compared to the LVF (L. Liu & Ioannides, 2010). Most recently, we showed that sex-categorisation of human faces is more accurate for UVF face targets than for LVF face targets (Quek & Finkbeiner, 2014a). Participants in this study were also able to extract the sex information carried by nonconsciously presented faces to a greater extent when the masked faces appeared in the upper-hemifield compared to the lower-hemifield. Moreover, nonconscious face-processing seemed to depend on the allocation of spatial attention when the faces appeared in the LVF, but not when they appeared in the UVF. Taken together, these results suggest that face-processing may be more efficient in the upper-hemifield than the lower-hemifield.

The notion of an upper-hemifield advantage for face-perception resonates with Previc’s (1990) proposal that vision in the UVF has become specialised to support the visual search and object recognition processes often required in extrapersonal space. After all, the UVF is where we most frequently encounter faces as we move through the world, and recognising the sex, identity, and expressions of these faces are undeniably some of the most critical forms of object recognition the brain performs. However, while the possibility that object recognition in general may be better above-fixation is certainly intriguing, it must be acknowledged that, to date, the UVF advantage for recognition documented in the literature is largely restricted to tasks involving face-perception (Coolican, Eskes, McMullen, & Lecky, 2008; Felisberti & McDermott, 2013; Kessler & Tipper, 2004; L. Liu & Ioannides, 2010; Quek & Finkbeiner, 2014a). Only a handful of studies have examined vertical asymmetry effects for objects other than faces, the findings of which are largely equivocal (Chambers et al., 1999; Darker & Jordan, 2004; Goldstein & Babkoff, 2001; Hagenbeek & Van Strien, 2002; Schwartz & Kirsner, 1982). As such, it is not yet established whether the UVF advantage for faces is stimulus-specific—a not unreasonable possibility given the relatively special status faces enjoy within the visual system (Farah, 1996; Farah, Wilson, Drain, & Tanaka, 1998; Kanwisher, 2000; Kanwisher, McDermott, & Chun, 1997).

Our purpose in the present paper was to establish whether the UVF advantage we have previously reported for face-sex categorisation would extend beyond face stimuli. To this end, we asked whether vertical hemifield presentation would modulate the perception of a nonface object - human hands. Hands are an ideal stimulus with which to pursue this line of enquiry, since they can also serve as the basis for sex-judgments (Gaetano, van der Zwan, Blair, & Brooks, 2014; Kovács et al., 2006). We are thus able to retain the sex-categorisation task we have used previously to demonstrate an upper-hemifield advantage for face stimuli (Quek & Finkbeiner, 2014a). To examine the effect of vertical hemifield presentation on hand-sex categorisation, we asked participants to identify the sex of a consciously presented hand image that appeared either immediately above or below central fixation on each trial. Participants indicated their sex-categorisation response by reaching out to touch one of two response panels positioned to the left and right of the computer monitor. We manipulated whether participants attended to the spatial location of the hand by way of a peripheral precue procedure in which, 100ms before target onset, a spatial cue briefly appeared in a location vertically adjacent to either the upper or lower stimulus position. Peripheral precues have been argued to capture covert attention in a largely automatic fashion (Jonides, 1981; Müller & Rabbitt, 1989; Pestilli & Carrasco, 2005; Pestilli, Viera, & Carrasco, 2007; Posner, 1980), and indeed we ourselves have shown the procedure to yield strong cue validity effects (Finkbeiner & Palermo, 2009; Quek & Finkbeiner, 2013, 2014a).

If object recognition processes supporting sex-categorisation really do enjoy an advantage in the upper-hemifield, there are two ways this might be reflected in our experiment. First, hand sex-categorisation itself might be more accurate and/or efficient in the UVF compared to the LVF. Second, sex-categorisation of hand stimuli in the upper- and lower-hemifields may be differentially modulated by attention, just as we have seen for face-sex categorisation (Quek & Finkbeiner, 2014a). That is, if object recognition processes are superior in the UVF, then on the assumption that covert attention will provide the most aid to the least privileged locations (Carrasco, Giordano, & McElree, 2004; Carrasco & Yeshurun, 1998; Yeshurun & Carrasco, 1998, 1999), our manipulation of focussed spatial attention should modulate target processing more in the (disadvantaged) lower-hemifield than the (advantaged) upper-hemifield. Indeed, it may be the case that object recognition is so efficient in the UVF that responses to targets in this location do not benefit (or suffer) from shifts in spatial attention at all (see Quek & Finkbeiner, 2014a). To anticipate our results, we observed that while participants’ accuracy and efficiency in categorising the sex of hand images did not differ between the vertical hemifields, the sex-categorisation response was indeed more sensitive to the effects of spatial attention when the target appeared in the LVF than the UVF. That is, spatial attention modulated the sex-categorisation response for lower-hemifield hand targets, but not upper-hemifield hand targets.

## Materials & Methods

### Ethics

Experimental protocol was approved by the Human Research Ethics Committee of Macquarie University. All procedures were in compliance with the guidelines laid out in the National Health and Medical Research Council (NHMRC) National Statement (2007). We obtained informed written consent from all participants described in this study.

### Participants

Thirty-six Macquarie University undergraduate students (22 females, 14 males) completed the experiment in exchange for course credit or financial compensation. Participants ranged in age from 18 to 41 years (*M*_Age_ = 20.78; *SD*_Age_ = 3.91 years). All participants had normal or corrected-to-normal vision, and were identified as strong right handers using the Edinburgh Handedness Inventory (Oldfield, 1971).

### Stimuli

**Figure 1.**
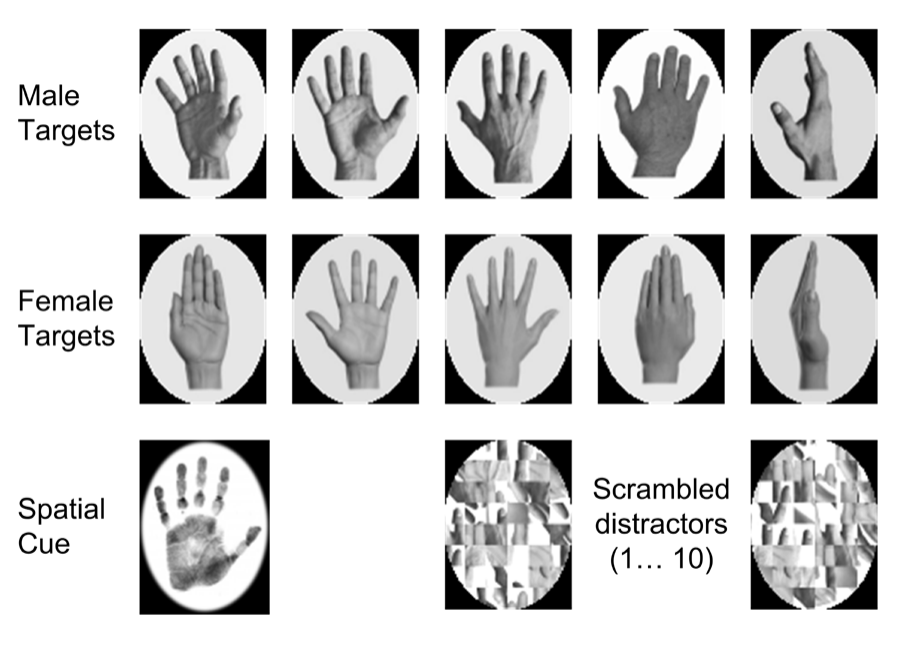
Targets were five male and five female hand targets in various poses. Distractors were 10 randomly generated scrambled images of male and female hands. We adjusted the low level properties of all targets and distractors so that their mean luminance was comparable. The spatial cue was a hand print image that contained no obvious sex information.

Figure 1 presents the stimuli used during the experiment. We chose these stimuli from a set of 46 greyscale pictures of hands (23 male, 23 female) taken from the internet and in-house sources. Thirty-two anonymous respondents (24 female, 8 male) aged between 18 and 34 years rated each of these 46 images according to how male the hand appeared to them (1 = *not at all male*; 5 = *extremely male*). We then selected as targets five images which were consistently rated at the top of the scale (e.g., 31/32 respondents rated the fourth male target as *extremely male*); and five images consistently rated at the bottom of the scale (e.g., 29/32 respondents rated the first female target as *not at all male*). The final set of 10 targets thus included five male and five female greyscale hands shown in various poses on a white oval background. Distractors were 10 greyscale scrambled images created from sample male and female hand images that did not appear as targets. We used the SHINE toolbox written for Matlab to adjust all target and distractor items so their mean luminance and contrast values were comparable (Willenbockel, Sadr, Fiset, Horne, Gosselin, & Tanaka, 2010). The spatial cue was a greyscale handprint on a white background and was not discernibly male or female. All stimuli appeared on a black background and subtended 1.53° × 2.13° of visual angle from a viewing distance of 1,050 mm.

### Apparatus

The participant sat before a rigid table with an LCD monitor positioned 850mm from the front edge. Left and right wooden response panels (270 × 90 mm) were fixed 750 mm apart, 500 mm from the table edge. The participants classified the sex of the target images by using their right (dominant) hand to reach out to touch one of the response panels, with the correct touch position for male and female targets counterbalanced across participants. A Polhemus Liberty electromagnetic tracking system recorded the reaching response in *xyz* space by sampling the position of a small sensor affixed to the tip of the right index finger at a rate of 240 Hz. We used custom software written in Presentation® to present stimuli and interface with the motion capture system.

### Procedure

**Figure 2.**
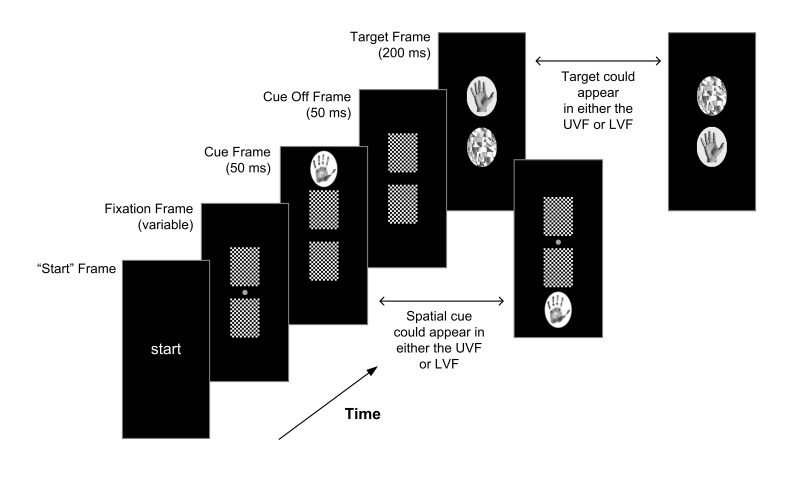
Visual trial structure. Participants began each trial by viewing a “start” screen. This remained present until they initiated the trial sequence by moving their right index finger into the “start” region at the front of the table. The initial fixation frame consisted of two forward chequerboard masks, the duration of which varied trial to trial. We used a briefly presented peripheral cue (50 ms) to capture spatial attention to either the upper or lower hemifield. After an inter-stimulus interval of 50 ms, the target then appeared for 200 ms in either the top or bottom panel, accompanied by a scrambled distractor opposite. There were 40 trials per block. Participants completed 10 experimental blocks preceded by two initial practice blocks (discarded prior to analysis).

Figure 2 shows the visual trial structure for this experiment. Each trial was preceded by a “Start” screen that remained onscreen until the participant initiated the trial proper by moving the Liberty sensor attached to their right index finger into a “start” region aligned with the body midline at the front of the testing table. Each frame following this consisted of two identically sized panels vertically displaced around a central fixation point (1.26° of visual angle from fixation to the centre of each panel). First, participants saw a fixation frame of two identical chequerboard forward masks. We varied the duration of this fixation frame to increase participants’ uncertainty regarding target onset. Next, an exogenous spatial cue appeared for 50 ms either immediately above or below the top or bottom panel respectively (see Figure 2). At cue offset, the forward masks remained onscreen for another 50 ms before being replaced by the critical target image and a randomly selected distractor (200 ms duration). A blank screen then remained until the participant completed their response by touching one of the two target panels, after which they received visual feedback on their classification (“…Correct…” or “… WRONG…”). We used a stimulus onset asynchrony (SOA) of 100 ms between the cue and target to maximise the benefit of exogenously captured covert attention, known to occur around 80-120 ms following cue onset (Müller & Rabbitt, 1989; Nakayama & Mackeben, 1989).

We trained participants to initiate their classification reaching movements in response to an imperative auditory go-signal. On each trial, participants heard a train of three ascending beeps and had to begin moving in synchrony with the third beep in the series. We then varied the position of this third beep in time relative to target onset such that the target-to-beep SOA on any given trial could be 0 ms (40% probability), 150 ms (40% probability), or 250 ms (20% probability). By requiring participants to begin moving on the basis of an auditory go-signal, rather than simply in response to the target’s appearance, we ensured a wide range of target-viewing times across the whole experiment. This was important insofar as we wanted to observe any experimental effects as they unfolded during stimulus-processing time (see Data Preparation). We defined Movement Initiation Time (MIT) on each trial as the time in Milliseconds from target onset until the right index finger’s tangential velocity reached 10 cm/second. To ensure participants did indeed synchronise their movement onset with our auditory go-signal, we required MIT latency on each trial to fall within a 300 ms response window around the third beep (-100 ms to 200 ms). We aborted any trials on which MIT fell outside this window and cached them for representation at the end of each block, and also presented participants with negative feedback (e.g., “… Too slow!” accompanied by a loud buzz). Over the course of the full experiment then, MIT latencies for each participant ranged from -100 ms to 450 ms. Importantly, although we maintained strict control of when participants should begin their reaching movement, once initiated the reaching response was quite unhurried. Participants had over three seconds after movement initiation in which they were free to change, correct, and complete their classification response.

### Design

We used a 2 × 2 fully-crossed within subjects design with the factors Visual Field (Upper or Lower) and Cue Validity (Valid or Invalid). Each of the 10 targets appeared in each of these four experimental conditions once per block and the full experiment consisted of 10 experimental blocks. There were two practice blocks at the start of the experiment; we excluded data from these blocks from later analyses.

### Analysis Methods

#### Data Preparation

Two participants produced accuracy rates less than 70% and were thus discarded from further analyses. We examined the remaining 34 participants’ data to remove all trials on which movement error occurred (i.e., moving too early or too late; 10.2% of all trials). As described above, on each trial we sampled the *xyz* coordinates of the finger’s position in space every 4 ms, from start of each trial until the participant touched one of the two response panels. To prepare each raw trajectory for analysis, we first determined the movement onset on that trial (i.e., MIT, or the point in time when the finger’s tangential velocity reached 10 cm/second). We then calculated our dependent measure at each of the 450 samples between the points corresponding to 100 ms before movement onset and 1,700 ms after movement onset. It was important to include the 100 ms leading up to movement onset so as to ensure we considered the very start of the reaching response on each trial. For any trials on which the participant touched a response panel less than 1,700 ms after their movement began, we simply repeated the xyz coordinates from the final sample to make up the full 450 samples for that trial.

We used *x*-velocity as our dependent measure, defined as the finger’s velocity along the left-right or *x* axis. Because participants indicate their sex-categorisation decision along this dimension (e.g., left for male; right for female), *x*-velocity reflects the finger’s velocity in the correct direction. *x*-velocity is a signed value, in that positive values indicate the finger is moving towards the correct response panel, and negative values that the finger is heading away from the correct response panel (i.e., in the incorrect direction). When considered as a continuous dataset, *x*-velocity values essentially provide a moment-to-moment index of the participant’s response certainty over the course of the reaching movement. That is, the faster the finger moves in the correct direction (i.e., the higher *x*-velocity is), the more certain the participant must be regarding their classification decision. To understand how *x*-velocity reflects response certainty, consider the following example. Say participants are engaged in a lexical decision task in which they must reach left in response to pseudoword targets and right in response to word targets. Just as RTs are typically shorter for high frequency words (e.g., *follow*) than for low frequency words (e.g., *beckon*), here we would expect participants’ reaching movements to be more efficient (i.e., head in the correct direction sooner) in the former condition (for a detailed discussion of reaching trajectory analyses, see Finkbeiner, Coltheart, & Coltheart, 2014). We used a modified version of the Orthogonal Polynomial Trend Analysis (OPTA) procedure (Karayanidis, Provost, Brown, Paton, & Heathcote, 2011; Woestenburg, Verbaten, & Slangen, 1983) to smooth each *x*-velocity profile individually (for details of this procedure, see Finkbeiner et al., 2014; Quek & Finkbeiner, 2013, 2014a). As a final step, we calculated a single value for each trial, termed initial *x*-velocity, by averaging *x*-velocity values across the first 200 ms following target onset. These initial *x*-velocity values were subjected to the statistical analyses described below.

#### Statistical Analyses

We analysed participants’ accuracy and initial *x*-velocity data using custom software written in R (www.r-project.org). We conducted linear mixed-effects modelling (LMM; Baayen, Davidson, & Bates, 2008; Bates, 2005) using the lmer4 package written for R (http://lme4.r-forge.r-project.org; Bates, Maechler, & Bolker, 2011). The advantage of using LMM over traditional ANOVA techniques is that LMM analysis enables the researcher to consider both fixed and random effects simultaneously. LMM analysis has also been argued to suffer less severe loss of statistical power when an experimental design becomes unbalanced as a result of missing data (see Pinheiro & Bates, 2000). Since LMMs incorporate individual trial data (rather than averaged conditional data), this analysis method can retain participant data that a mixed-model ANOVA may be obliged to exclude entirely due to a low number of trials in a particular design cell (Kliegl, Masson, & Richter, 2010; Kliegl, Wei, Dambacher, Yan, & Zhou, 2011).

Generalised LMM has been argued to be the most appropriate analysis for discrete accuracy data (Dixon, 2008). We modelled our data using an incremental model comparison procedure in which we evaluated the reliability of each effect by examining which model fit the data better—one that included the effect of interest or one that did not. The preferred model was that which minimised the goodness-of-fit statistics AIC and BIC and maximised the Log Likelihood value (Akaike, 1974; Schwarz, 1978). Below we report the results of this Likelihood ratio test, and where appropriate, the coefficients, standard errors (*SE*), and *t*-values for the terms included in the final model selected. Our criterion for significance for individual fixed effects was an absolute *t* ratio of 2.0, as per Kliegl et al. (2010). Although the degrees of freedom are not known exactly in LMM, the very large number of observations in the datasets used here and elsewhere mean that the *t* distribution converges to the normal distribution. Thus, the criterion cutoff of *SE* = 2.00 corresponds well to the .05 significance criterion (see Finkbeiner et al., 2014; Kliegl et al, 2010; Masson & Kliegl, 2013; Quek & Finkbeiner, 2013, 2014a).

## Results

### Accuracy

**Figure 3.**
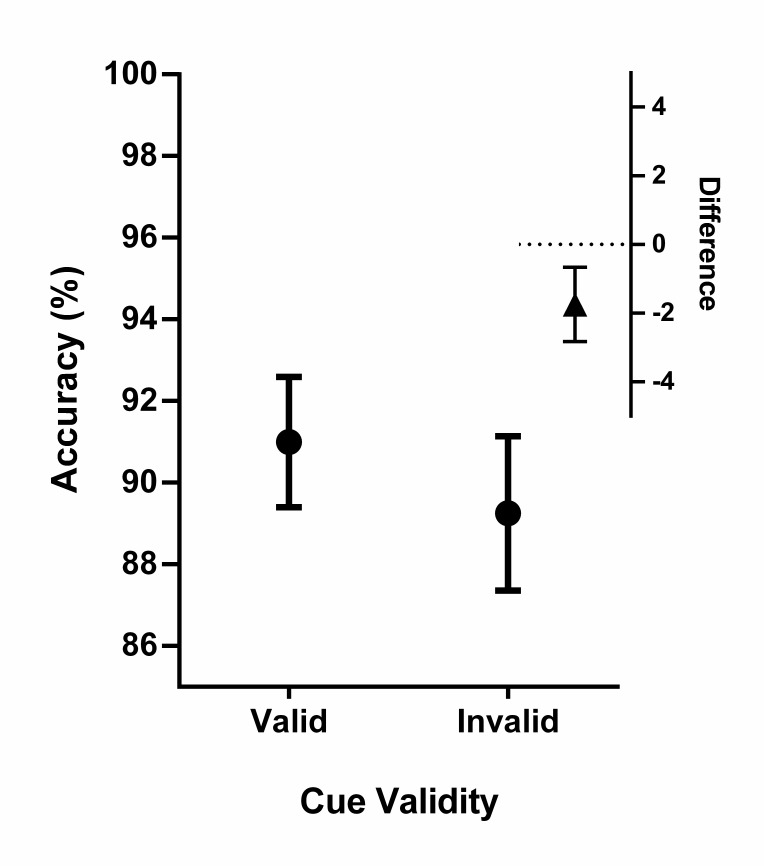
Main: Hand sex-categorisation accuracy rates as a function of Cue Validity. Binomial LMM analyses indicated participants were significantly more likely to classify the target correctly on validly cued trials than on invalidly cued trials. Error bars represent within-subjects 95% confidence intervals (WSCIs). Visual Field had no effect on accuracy rates and is not depicted here. I nset: Since there is no rule-of-eye for interpreting overlap between WSCIs (see Cumming & Finch, 2005), we here depict the 95% CI around the mean of the differences (valid–invalid). Since the CI around the mean difference score excludes zero, we must reject *H*_0_ and concede that accuracy rates were higher for validly cued trials compared to invalidly cued trials.

The overall sex-categorisation accuracy rate was 90%. Accuracy rates close to ceiling are typical of the reach-to-touch paradigm, since unlike button press tasks, this response measure allows participants to correct their initial decision about the target before making their final choice (e.g. Quek & Finkbeiner, 2013, 2014a). Despite these high accuracy scores, we were still able to observe experimental effects of interest in participants’ accuracy rates. We analysed the binomial accuracy data using an LMM which included Participant as a random factor. Using the model comparison procedure described above, we then verified that including the fixed effect of Cue Validity significantly improved the fit of the model, χ^2^(1) = 11.97, *p* < .001. Participants were significantly less likely[Fn FN1]^1^ to classify the target correctly on invalidly cued trials than on validly cued trials (*b* = -0.20, *SE* = 0.06, *z* = -3.46, *p* < .001, see Figure 3), indicating our spatial cueing procedure was effective in manipulating participants’ spatial attention (*M*_VAL_ = 91%, *M*_INVAL_ = 89%, Cohen’s *d* = .35). By contrast, neither the fixed effect of Visual Field, χ^2^(1) = .12, *p* = .729 (*M*_UVF_ = 90%, *M*_LV_ = 90%; Cohen’s *d* = .04), nor the interaction between Visual Field and Cue Validity, χ^2^(1) = .00, *p* =.982, significantly improve the model; these terms were thus excluded from the final model for accuracy data[Fn FN2]^2^.

### Initial *x*-velocity

#### Analyses collapsed across target-viewing time

**Figure 4.**
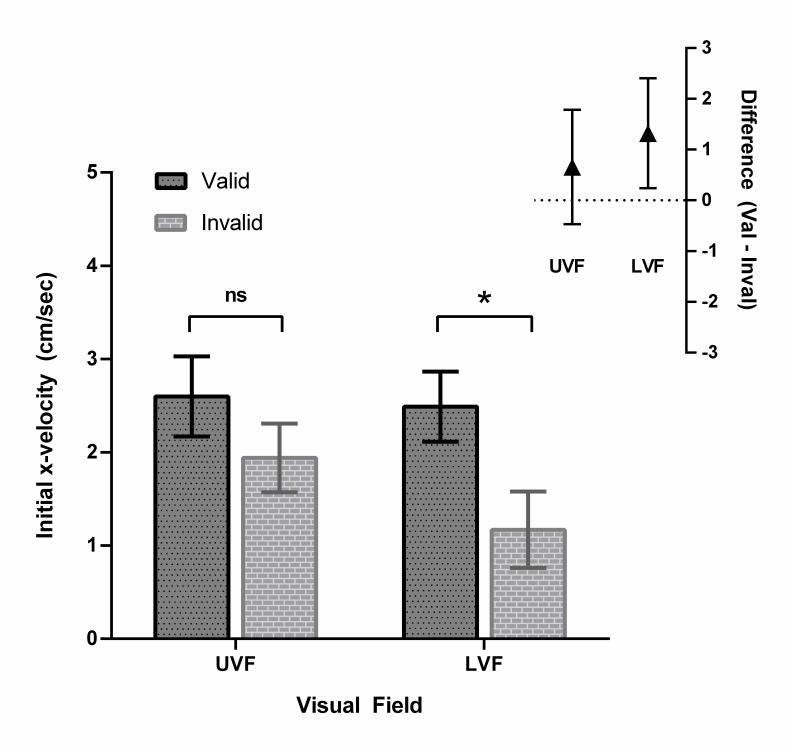
Main: Initial *x*-velocity as a function of Cue Validity and Visual Field. We followed up on the significant interaction between these factors by conducting paired *t*-tests between the valid and invalid cue conditions for each visual field. The effect of Cue Validity was reliable in the LVF, but not the UVF (*p < .05; corrected using False Discovery Rate). Error bars represent 95% WSCIs. Inset: 95% CIs around the mean of the differences (valid– invalid) for each visual field. The CI around the mean difference for the LVF excludes zero, indicating that initial *x*-velocity was significantly higher for valid compared to invalid trials in the LVF. In contrast, the CI around the mean difference for the UVF captures zero, indicating the difference between valid and invalid trials was not reliable in the UVF.

We submitted the 11,644 initial *x*-velocity values to LMM analysis which included random intercepts for Participant. We used the model comparison procedure described above to verify that the inclusion of Cue Validity, χ^2^(1) = 141.72, *p* < .001, Visual Field, χ^2^(1) = 21.06, *p* < .001, and the interaction between these factors, χ^2^(1) = 14.48, *p* < .001, all improved the fit of the model. Thus, we included all three terms in our final model of initial *x*-velocity. As may be seen in Figure 4, there was a main effect of Cue Validity (*b* = -.68, *SE* = 0.12, *t* = -5.76, Cohen’s *d* = .34), whereby initial *x*-velocity was significantly higher on validly cued (M = 6.60 cm/second) compared to invalidly cued trials (*M* = 5.25 cm/second). In contrast, the main effect of Visual Field was not significant (*b* = -.07, *SE* = 0.12, *t* = -.59, Cohen’s *d* = .14), however we did observe a significant interaction between the factors (*b* = -0.64, *SE* = 0.17, *t* = -3.81). As may be seen in Figure 4, the cueing effect was stronger when targets appeared in the LVF compared to the UVF. Follow-up paired *t*-tests between the valid and invalid cue conditions within each vertical hemifield indicated the cueing effect was reliable in the LVF, *t*(33) = 2.49, *p* = .036, Cohen’s *d* = .43, but not the UVF, *t*(33) = 1.19, *p* = .244, Cohen’s *d* = .17, (*p* values corrected using False Discovery Rate, FDR)[Fn FN3]^3^.

#### Analyses taking target-viewing time into account

**Figure 5.**
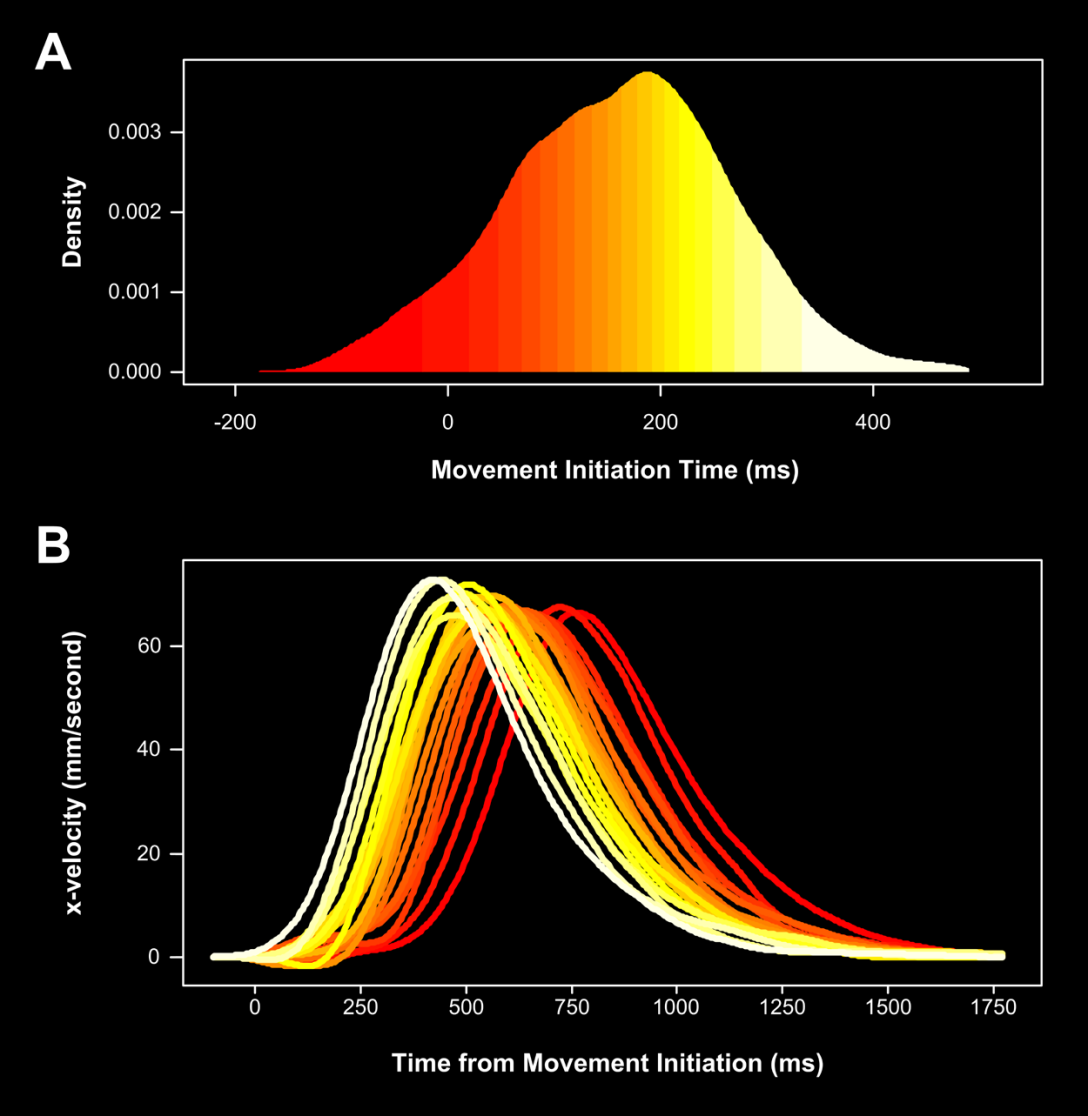
The effect of target-viewing time on initial *x*-velocity. (A) The movement initiation time (MIT) latency distribution. We grouped MIT latencies into 20 incremental quantiles (i.e., semideciles), represented here by the colour gradient (dark/red colours = short MIT latencies; yellow/pale colours = long MIT latencies). (B) Averaged *x*-velocity profiles as a function of MIT Quantile. It is clear that the *x*-velocity profiles of reaching trajectories initiated very soon after target onset (i.e., short MITs) tend to peak much later in time than those initiated after a long target-viewing time (i.e., long MITs). In other words, the longer participants view the target before commencing their reaching response, the more efficiently they are able to classify the target.

A unique advantage of the version of the reach-to-touch paradigm used here is that it enables the experimenter to examine not only the magnitude, but also the timecourse of experimental effects (for an extended discussion, see Finkbeiner et al., 2014). By requiring participants to initiate their classification movement in response to an auditory go-signal whose onset varied in time with respect to the target, we were able to examine how target-viewing time modulated our experimental effects reflected in initial *x*-velocity. The OPTA procedure discussed above (see also Finkbeiner et al., 2014; Quek & Finkbeiner, 2013, 2014a) allowed us to take account of the relationship between target-viewing time and *x*-velocity by treating MIT latency as a covariate during data-smoothing. Figure 5 shows the strong effect target-viewing time (i.e., MIT latency) had on the way the reaching response itself unfolded. Here we have grouped the MIT latencies into 20 quantiles, or semideciles (i.e., the shortest 5%, then the next shortest 5%, and so on - plotted in Figure 5a) and calculated the average *x*-velocity profile for each of these MIT Quantiles (plotted in Figure 5b). Looking at this figure, it is evident that the longer participants viewed the target before initiating their classification response (i.e., the greater their MIT latency was), the more efficient their reaching movement was when it began (i.e., the faster their finger headed in the correct direction).

**Figure 6.**
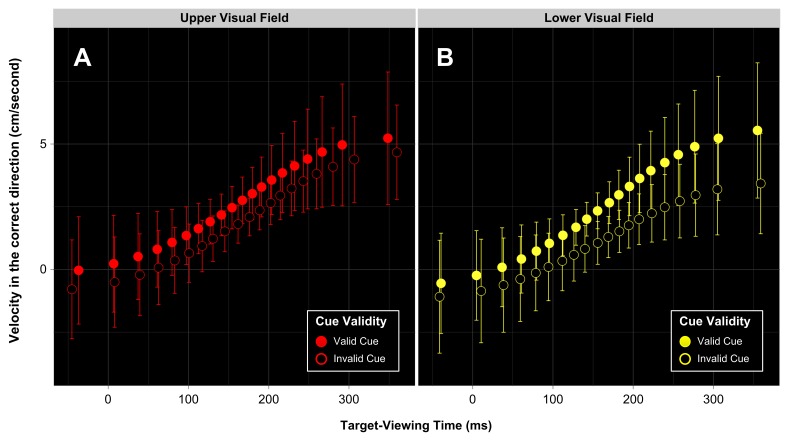
Initial *x*-velocity (velocity averaged across the first 200 ms of reaching movement) as a function of Visual Field, Cue Validity, and Target Viewing-Time [i.e., movement-initiation time (MIT) Quantile]. The slope of these profiles indicates that the longer participants view the target before commencing their reaching movement, the faster their finger heads in the correct direction during the reaching response itself. Initial *x*-velocity is higher on validly cued trials compared to invalidly cued trials. Error bars are 95% within-subject confidence intervals (WSCIs) – note that the overlap of these error bars should not be interpreted by eye (refer to Figure 7 for difference score plots).

To analyse initial *x*-velocity as a function of target-viewing time, we conducted LMM analysis which included MIT Quantile as a factor. We began with a model which included random intercepts for Participant and random slopes between Participant and Quantile. We then used model comparison to confirm that the fixed effects of MIT Quantile, χ^2^(1) = 14.47, *p* < .001, Cue Validity, χ^2^(1) = 228.21, *p* < .001, and Visual Field, χ^2^(1) = 33.91, *p* < .001, all improved the model’s fit. As may be seen in Figure 6, initial *x*-velocity tended to increase with MIT Quantile (*b* = 0.29, *SE* = 0.07, *t* = 4.12). Initial *x*-velocity was also higher, on average, for validly cued trials than for invalidly cued trials (*b* = -0.57, *SE* = 0.14, *t* = -4.19), and when the target appeared in the UVF compared to the LVF (*b* = -0.49, *SE* = 0.14, *t* = -3.58). Regarding interactions, rather than evaluate each possible interaction given by our multifactorial design, we then restricted ourselves to examining the interactions which could reveal information about the timecourse of our experimental effects (i.e., those involving MIT Quantile). First, we verified that including the two-way interaction between Cue Validity × MIT Quantile improved the model’s fit, χ^2^(1) = 12.61, *p* < .001. In contrast, the interaction between Visual Field × MIT Quantile did not improve the model, χ^2^(1) = 0.79, *p* = .375. Importantly, however, the critical three-way interaction between Cue Validity × MIT Quantile × Visual Field did significantly improved how well the model fit the data, χ^2^(1) = 40.20, *p* < .001^4^.

**Figure 7.**
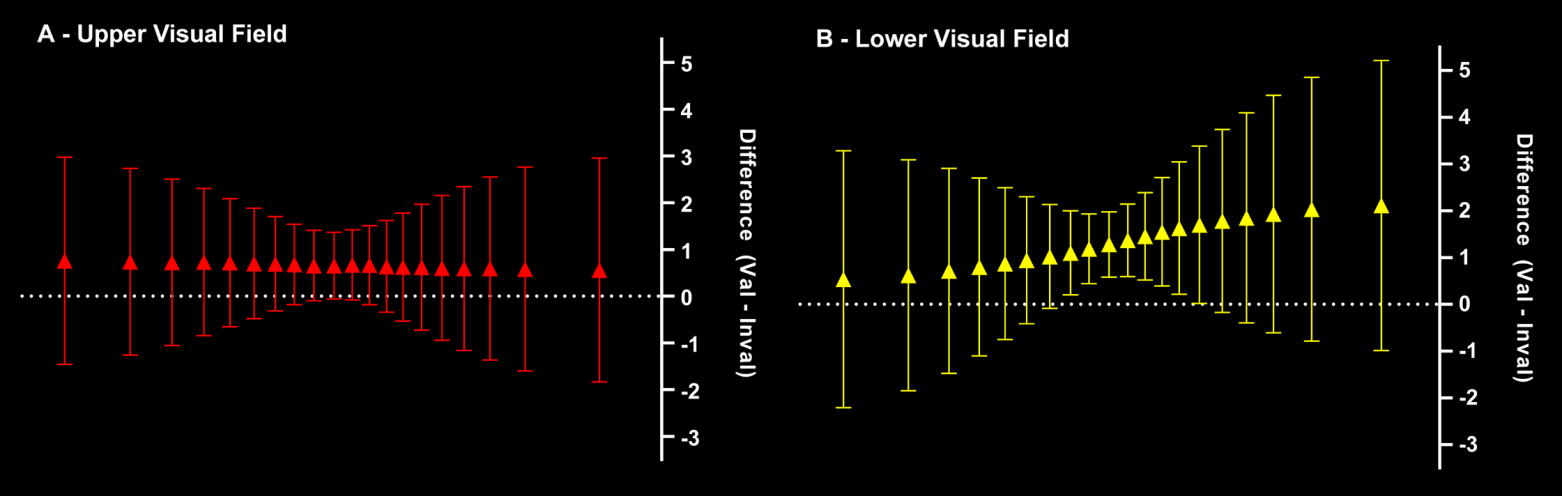
Difference scores (valid – invalid) for the (A) upper visual field (UVF) and (B) lower visual field (LVF). Datapoints represent the size of the validity effect at each movement-initiation time (MIT) Quantile, with a net validity effect of zero given by the dashed line. A 95% within-subject confidence interval (WSCI) around the mean difference that excludes zero indicates initial *x*-velocity to be reliably higher for valid than invalid trials at that MIT Quantile. A comparison of the upper and lower panels shows that the validity effect in the UVF was not reliable at any MIT Quantile, whereas a reliable validity effect in the LVF was present from the 8th to the 14th MIT Quantile.

To follow up on the nature of the three-way interaction depicted in Figure 6, for each Visual Field we computed a difference score (valid - invalid) at each MIT Quantile (see Figure 7). We then inspected each 95% confidence interval around each difference score to see whether it included zero or not. Using this comparison method, we observed that the validity effect was reliable from the 8th to 14th MIT Quantile in the LVF, but not at any MIT Quantile in the UVF^5^.

## General Discussion

The present study set out to determine whether the UVF advantage that has been observed for face-sex categorisation (Quek & Finkbeiner, 2014a) would extend to the categorisation of nonface objects. To this end, we used the same task and response paradigm as Quek and Finkbeiner (2014a) to examine how vertical hemifield would modulate participants’ ability to categorise the sex of human hands. The results we report suggest that hand-sex categorisation enjoys a subtle UVF advantage similar to that for faces (albeit somewhat less robust) (Quek & Finkbeiner, 2014a). Specifically, we observed no difference between the upper- and lower-hemifields in the accuracy or efficiency with which participants were able to classify the sex of visible hand targets. In contrast, hand-sex categorisation was strongly modulated by focussed spatial attention, in that participants’ reaching responses were both more accurate and efficient when spatial attention was captured to the target’s location. Critically, however, this effect of cue validity on categorisation efficiency was qualified by vertical hemifield, in that this manipulation of spatial attention had a larger and more reliable effect on the categorisation of targets presented in the LVF compared to the UVF. On the assumption that focussed spatial attention will provide the most aid to the least privileged locations (Carrasco et al., 2004; Carrasco & Yeshurun, 1998; Yeshurun & Carrasco, 1998, 1999), the finding that target classification responses were more sensitive to the effects of spatial attention in the LVF than the UVF suggests that sex-categorisation of human hands is less robust in the lower-hemifield. Conversely, the smaller and less reliable impact of spatial cueing on target categorisation in the UVF suggests the processes supporting hand-sex recognition are more efficient in this region of space.

Importantly, this differential benefit of attention on hand-sex categorisation in the UVF and LVF is highly consistent with our previous report that covert spatial attention modulates sex-categorisation of faces differently in the upper- and lower-hemifields (Quek & Finkbeiner, 2014a). In this previous work, we found that participants’ ability to extract sex information carried by nonconscious faces depended on the allocation of spatial attention in the LVF, but not in the UVF. In conjunction with the present findings then, this suggests that the lower-hemifield’s increased sensitivity to the effects of spatial attention in the context of sex-categorisation holds across two distinct stimulus types (i.e., faces and hands). That spatial attention improves the efficiency of processes supporting sex-categorisation in the LVF (but not the UVF) is particularly intriguing in light of previous work by Carrasco and colleagues which showed covert attention speeds information accrual to a greater degree at upper vertical meridian locations than lower (Carrasco et al., 2004). While the basis for these inconsistent findings is not yet clear, a potential explanation may lie in the different stimuli and tasks employed in these studies. Participants in Carrasco et al. (2004) performed an orientation discrimination task for Gabor stimuli presented in the periphery. A speed-accuracy tradeoff (SAT) analysis showed that information processing was significantly faster for LVF targets compared to UVF targets—an expected finding given that performance in this task is based on contrast sensitivity, which is known to be advantaged at below-fixation locations (Cameron et al., 2002; Carrasco et al., 2001; T. Liu et al., 2006). As a result, their manipulation of focussed attention modulated the rate of information accrual to a greater extent in the disadvantaged UVF, where there was room to observe an attentional effect.

In contrast to this relatively low-level visual discrimination task, sex-categorisation of hand and face images presumably depends on higher-level object recognition processes—processes which could very well exhibit a different pattern of vertical asymmetry effects in which the UVF is superior to the LVF. While on balance there have been very few investigations of vertical hemifield effects for higher level object stimuli, there is increasing evidence that face-processing is supported better in above-fixation locations compared to below (Coolican et al., 2008; L. Liu & Ioannides, 2010; Quek & Finkbeiner, 2014a). Only a handful of studies, however, have alluded to similar findings for nonface stimuli such as novel objects (Chambers et al., 1999), letters (Schwartz & Kirsner, 1982), and words (Goldstein & Babkoff, 2001). As such, the present results represent an important contribution to the study of vertical asymmetry in higher level object recognition. Importantly, while these findings do support the notion that the upper-hemifield may enjoy an advantage for object recognition in general (i.e., not just for faces) (Previc, 1990), clearly more rigorous investigation of vertical hemifield effects for nonhuman objects is required before this claim can be made.

In conjunction with previous findings (e.g., L. Liu & Ioannides, 2010; Quek & Finkbeiner, 2014a), the results presented here suggest that both face- and hand-sex recognition processes enjoy an advantage in the UVF compared to the LVF. What mechanism might account for this consistent pattern across two distinct stimulus types? We speculate on two possible accounts here. First, one possibility has to do with bias in participants’ voluntarily directed spatial attention. Biases in the distribution of spatial attention have been of increasing interest (e.g. Loughnane, Shanley, Lalor, & O’Connell, 2015), and could be particularly relevant in the present case. Specifically, a differential benefit of *exogenously captured* attention between the vertical hemifields could arise if participants’ *voluntarily directed* attention was biased towards one hemifield over the other. It could be the case, for example, that participants might have favoured the UVF (consciously or otherwise) by voluntarily directing spatial attention toward this hemifield even while maintaining central fixation. If this were the case, then an exogenous manipulation of spatial attention might well be expected to have little effect in the UVF, since processing in this region would already be facilitated by the allocation of voluntarily directed covert attention. Similarly, it stands to reason that if spatial attention were already directed to the UVF in some endogenous capacity, then an exogenous cue in the LVF should be especially effective in this relatively less attended region. Although a speculative possibility at this point, interestingly there is already some suggestion in the literature that there may indeed be an upward bias in spatial attention under certain conditions. For example, studies involving vertical line bisection (Bradshaw, Nettleton, Nathan, & Wilson, 1985; Drain & Reuter-Lorenz, 1996; van Vugt, Fransen, Creten, & Paquier, 2000), object matching (Chambers et al., 1999), and mental scene representation (Drummond & Tlauka, 2012) suggest that participants may preferentially attend to the upper half of space over the lower half (but see Rezec & Dobkins, 2004). We have pursued the possibility of an upward bias in voluntarily directed spatial attention in subsequent series of experiments in which we manipulate the predictability of target location (Quek & Finkbeiner, 2014b).

Second, it is also possible that the UVF advantage that we have observed with both faces and hands can be attributed to the possibility that sex-relevant cues are clearest in the top-most part for these two classes of stimuli. For example, Bruce et al. (1993) have shown that masking the top of faces (eyes and eyebrows) negatively affects accuracy rates in a sex-discrimination task. Similarly, it has also been observed that the length ratio from the 2nd to 4th digit in human hands is a sexual dimorphism (smaller in males; cf. Putz, Gaulin, Sporter, & McBurney, 2004). Because these sex-relevant cues have consistently appeared in the top-most half of the stimuli that we have used (faces and hands), it remains to be seen if our findings could be explained by local sex-relevant cues corresponding with the global position of the stimulus (upper visual field) to yield better sex-discrimination when these two cues are compatible. This intriguing possibility of a local-global compatibility effect deserves further investigation in future studies.

## Conclusion

The results reported here suggest that sex-categorisation of human hands exhibits a subtle upper-hemifield advantage. We found that the effects of spatial attention on this task were more pronounced in the LVF than in the UVF, just as for face-sex categorisation (Quek & Finkbeiner, 2014a). Taken together, these data suggest that the UVF advantage for sex-categorisation extends to both face and nonface stimuli. As such, the findings provide empirical support for Previc’s (1990) speculation that object recognition processes may enjoy an advantage in above-fixation locations. While it is not yet known what might underpin this superior performance in the UVF for sex-categorisation tasks, we have speculated here on two possible accounts, including the possibility of an upward bias in participants’ voluntarily directed spatial attention as well as the possibility that local sex-relevant cues (e.g., of 2nd to 4th digit ratio in human hands) correspond with global position (e.g., upper visual field) to yield an UVF advantage in sex-discrimination tasks.

## Footnotes

1 For binomial data, positive and negative coefficients obtained using LMM indicate increasing and decreasing probabilities, respectively (Baayen et al., 2008).

2 An identical analysis including the two excluded participants’ data yielded the same pattern of results. LMM results: Cue Validity (*b* = -.19, *SE* = 0.05, *z* = -3.51, *p* < .001).

3 An identical analysis including the two excluded participants’ data yielded the same pattern of results. LMM results: Cue Validity (*b* = -.66, *SE* = 0.12, *t* = -5.50); Visual Field (*b* = .07, *SE* = 0.12, *t* = 0.56); Cue Validity × Visual Field (*b* = -.45, *SE* = 0.17, *t* = -2.67). Follow-up *t*-test results: UVF, *t*(35) = 1.13, *p* = .268, LVF, *t*(35) = 1.92, *p* = .064.

4 An identical analysis including the two excluded participants’ data yielded the same pattern of results. LMM results: MIT Quantile (*b* = .26, *SE* = 0.07, *t* = 3.55); Cue Validity (*b* = -.67, *SE* = 0.14, *t* = -4.81); Visual Field (*b* = .33, *SE* = 0.14, *t* = 2.36). An identical model comparison procedure including the two excluded participants’ data verified that including the MIT Quantile × Visual Field interaction, χ^2^(1) = 15.59, *p* < .001, and Cue Validity × MIT Quantile × Visual Field interaction, χ^2^(1) = 12.05, *p* < .001, significantly improved how well the model fit the data. The two-way interaction between MIT Quantile × Cue Validity trended towards significance, χ^2^(1) = 2.93, *p* < .09.

5 An identical analysis containing the two excluded participants’ data produced a near identical pattern of results, with a reliable validity effect observed from the 8th to 12th MIT Quantile for the LVF, but not at any MIT Quantile for the UVF.
